# From *Polyalthia* to *Polyalthiopsis* (Annonaceae): transfer of species enlarges a previously monotypic genus

**DOI:** 10.3897/phytokeys.148.50929

**Published:** 2020-05-26

**Authors:** Bine Xue, Hong-Bo Ding, Gang Yao, Yun-Yun Shao, Xiao-Jing Fan, Yun-Hong Tan

**Affiliations:** 1 College of Horticulture and Landscape Architecture, Zhongkai University of Agriculture and Engineering, Guangzhou 510225, Guangdong, China; 2 Southeast Asia Biodiversity Research Institute & Center for Integrative Conservation, Xishuangbanna Tropical Botanical Garden, Chinese Academy of Sciences, Menglun, Mengla, Yunnan 666303, China; 3 College of Forestry and Landscape Architecture, South China Agricultural University, Guangzhou, China; 4 Guangdong Provincial Key Laboratory of Digital Botanical Garden, South China Botanical Garden, Chinese Academy of Sciences, Guangzhou 510650, China; 5 South China Botanical Garden, Chinese Academy of Sciences, Guangzhou 510650, Guangdong, China; 6 Center of Conservation Biology, Core Botanical Gardens, Chinese Academy of Sciences, Menglun, Mengla, Yunnan 666303, China

**Keywords:** Annonaceae, molecular phylogeny, morphology, *Polyalthia chinensis*, *Polyalthia verrucipes*, *
Polyalthiopsis
*

## Abstract

The genus *Polyalthiopsis* Chaowasku (Annonaceae) was a poorly known monotypic genus from Vietnam that was recently segregated from the highly polyphyletic genus *Polyalthia* s.l. The sister clade relationship between *Polyalthiopsis* and *Miliusa* was not well established in previous study. The phylogenetic position of two *Polyalthia* spp. from China, *P. chinensis* S.K.Wu ex P.T.Li and *P. verrucipes* C.Y.Wu ex P.T.Li, remains unresolved and is shown here to be phylogenetically affiliated with *Polyalthiopsis*. Phylogenetic analyses of six chloroplast regions (*mat*K, *ndh*F, *psb*A-*trn*H, *rbc*L, *trn*L-F and *ycf*1; ca.7.3 kb, 60 accessions) unambiguously placed *Polyalthia chinensis* and *P. verrucipes* in the same clade with *Polyalthiopsis floribunda* (PP = 1, MPBS = 97%); the entire clade is sister to *Miliusa* with weak to strong support (PP = 1, MPBS = 54%). *Polyalthia chinensis* and *P. verrucipes* share several diagnostic characters with *Polyalthiopsis floribunda*, including the raised midrib on the upper surface of the leaf *in vivo*, conspicuous foliar glands when dried, petiole with transverse striations when dried and axillary inflorescences. The two species differ from *Polyalthiopsis floribunda* in having fewer flowers per inflorescence, longer linear petals and two ovules per carpel. On the basis of the combined molecular phylogenetic and morphological data, we propose two new combinations, *Polyalthiopsis chinensis* (S.K.Wu ex P.T.Li) B.Xue & Y.H.Tan and *Polyalthiopsis verrucipes* (C.Y.Wu ex P.T.Li) B.Xue & Y.H.Tan. The protologue of *Polyalthia verrucipes* did not include a description of the flowers, which we provide here. An updated description for the genus *Polyalthiopsis* and a key to species in the genus *Polyalthiopsis* is also provided.

## Introduction

The genus *Polyalthia* Blume has historically been the source of considerable taxonomic confusion in Annonaceae due to its highly polyphyletic status (van Setten and Koek-Noorman 1992; Doyle and Le Thomas 1994; Doyle et al. 2000). Recent molecular phylogenetic studies have clarified generic circumscriptions and resulted in the segregation of disparate elements from the previously highly polyphyletic genus *Polyalthia* s.l., including removal of species now placed in several new genera–*Maasia* Mols & al. (Mols et al. 2008), *Huberantha* Chaowasku (Chaowasku et al. 2012 [as “*Hubera*”], Chaowasku et al. 2015); *Wuodendron* B.Xue, Y.H.Tan & T.Chaowasku (Xue et al. 2018) and *Polyalthiopsis* Chaowasku (Chaowasku et al. 2018); the transfer of species to *Fenerivia* Diels (Saunders et al. 2011), *Marsypopetalum* Scheff. (Xue et al. 2011), *Monoon* Miq. (Xue et al. 2012), *Goniothalamus* (Blume) Hook. f. & Thomson (Tang et al. 2013), *Meiogyne* Miq. (Xue et al. 2014) and *Wangia* X.Guo & R.M.K.Saunders (Xue et al. 2016). The circumscription of *Polyalthia* s.str. was consequently redefined (Xue et al. 2012).

Amongst the newly segregated genera, *Polyalthiopsis* Chaowasku is a poorly known monotypic genus from Vietnam (Chaowasku et al. 2018). The type species *Polyalthiopsis floribunda* is known from only two field collections (*Poilane 10052*, P, A, BO, CMUB, HN, K, L, P; and *Chaowasku 128*, CMUB). The species was first collected in 1924 and described under the name *Polyalthia floribunda* Jovet-Ast (Jovet-Ast 1940). I.M. Turner (2016) subsequently transferred the name to *Huberantha*. Ninety years after the first collection, Chaowasku collected this species again in 2014 and was able to sequence it for a phylogenetic study. It was shown not to be congeneric with *Huberantha* and was instead retrieved (without statistical support) as sister to *Miliusa*, leading Chaowasku et al. (2018) to erect a monotypic genus, *Polyalthiopsis* Chaowasku, to accommodate it. With only one *Polyalthiopsis* species and limited DNA regions used in the phylogenetic study, the sister relationship between *Polyalthiopsis* and *Miliusa* was not well established. It is also difficult to identify important diagnostic characters for *Polyalthiopsis* with only one flowering collection and a single monocarp available for taxonomic comparison.

Several species names remain unresolved in *Polyalthia* and await assignment to specific genera (Xue 2013; Xue et al. 2012), including the Chinese endemics *Polyalthia chinensis* S.K.Wu ex P.T.Li and *P. verrucipes* C.Y.Wu ex P.T.Li. As with *P. floribunda*, these two species are represented by very few collections and lack adequate flowering and fruiting descriptions.

The name *Polyalthia chinensis* was published in 1976, based on a flowering collection (*Qinghai-Xizang Exped. 74-4451*, KUN & PE) from Mêdog, Xizang, China, in 1974 (Li 1976; Li and Gilbert 2011). A second sterile specimen was subsequently collected in 1983 (*B. S. Li & S. Z. Cheng 2668*, PE). Until now, the species was only represented by these two collections.

The name *Polyalthia verrucipes* was published in 1976, based on a fruiting collection (*C.W. Wang 76321*, IBSC, NAS, A, PE) from Menghai, Yunnan, China, in 1936 (Li 1976; Li and Gilbert 2011). A second collection with immature fruits was collected in 1957 (*Sino-Russia Exped. 9527*, KUN & PE). Although Hou and Li (2007) cited further collections (*S. K. Wu 1345*, *1375*, KUN; *X. L. Hou 112*, CANT, IBSC; and *T. X. Sun 200037*, CANT), we failed to locate those specimens in the cited herbaria.

The relationship between these two species has been controversial. Both species are represented by very few collections, with *P. chinensis* lacking fruiting specimens and *P. verrucipes* lacking flowering specimens, rendering morphological comparison problematic. Based on the foliar glands and leaf venation, Hou and Li (2007) regarded the name *P. chinensis* as synonymous with *P. verrucipes*, although this treatment was rejected by Li and Gilbert (2011) in the *Flora of China* without explanation. Li and Gilbert (2011) included identical floral descriptions in their treatment of *P. verrucipes* and *P. chinensis*, but with no clear indication of the source of this information, casting some doubt over the floral description of *P. verrucipes*.

With limited morphological characters, especially the lack of flowers in *P. verrucipes* and the limited material available, the relationship between *P. chinensis* and *P. verrucipes* and their taxonomic placement has never been resolved. We therefore, carried out several field explorations to search for these two species. This resulted in new collections of *Polyalthia verrucipes*, including flowers, enabling clarification of the relationship between *P. chinensis* and *P. verrucipes*, as well as their phylogenetic position. As a consequence, we were able to enlarge the poorly known genus *Polyalthiopsis*, supplementing available descriptions and providing better support for its sister relationship.

## Phylogenetic analysis

### Taxon and DNA region sampling

Two accessions of *Polyalthia chinensis* (*B. S. Li & S. Z. Cheng 2668*, PE; and *Qinghai-Xizang Exped. 74-4451*, KUN), as well as two accessions of *Polyalthia verrucipes* (*Sino-Russia Exped. 9527*, PE; and *Y.H. Tan MH1603*, IBSC) were sampled and integrated with data of 56 Annonaceae accessions from previous datasets (Chaowasku et al. 2018; Guo et al. 2014; Xue et al. 2016, 2018). The final dataset comprised a total of 60 accessions of Annonaceae representing all major clades in the family, including 44 accessions representing 29 genera from subfam. Malmeoideae, 12 accessions representing 11 genera from subfam. Annonoideae, three species from subfam. Ambavioideae and one species of *Anaxagorea* A. Saint.-Hilaire. (subfam. Anaxagoreoideae). For Miliuseae, representatives of all currently accepted genera were included.

Six chloroplast DNA regions (*matK*, *ndhF*, *rbcL*, *psbA-trnH* and *trnL-F* and *ycf1*) were sequenced for the above-mentioned four collections of *Polyalthia chinensis* and *P. verrucipes*. The samples, localities and GenBank accession numbers are listed in Appendix I.

### DNA extraction, amplification and sequencing

Genomic DNA was extracted from herbarium materials using a modified cetyl trimethyl ammonium bromide (CTAB) method (Doyle and Doyle 1987). A single amplification protocol was used for amplification of the chloroplast regions: template denaturation at 94 °C for 5 min, followed by 35 cycles of denaturation at 95 °C for 30 sec; primer annealing at 50 °C for 1 min; and primer extension at 72 °C for 1 min, followed by a final extension step at 72 °C for 10 min. The primers used to amplify the *psbA-trnH* intergenic spacer were psbAF (Sang et al. 1997) and trnH2 (Tate and Simpson 2003); other primers are the same as those used by Thomas et al. (2012). PCR products were visualised using agarose gel electrophoresis. Successful amplifications were purified and sequenced on an Applied Biosystems 3730xl DNA Analyzer at Sangon Biotech (Shanghai) Co. Ltd., Guangzhou, China.

### Alignment and phylogenetic analyses

Sequences were assembled and edited using Geneious ver. 5.4.3 (Drummond et al. 2010) and pre-aligned with the MAFFT (Katoh et al. 2002) plugin in Geneious using the automatic algorithm selection and default settings and, subsequently, manually checked and optimised. An inversion of 15 positions in *psbA-trnH* was identified and reverse complemented in the alignment, following a strategy previously applied by Pirie et al. (2006), to retain substitution information in the fragments.

Maximum parsimony (MP) analyses of the seven combined regions were conducted using PAUP ver. 4.0b10 (Swofford 2003). All characters were weighted equally and gaps treated as missing data. The most parsimonious trees were obtained with heuristic searches of 1,000 replicates of random stepwise sequence addition, tree bisection-reconnection (TBR) branch swapping with no limit to the number of trees saved. Bootstrap support (BS) was calculated following Müller (2005), with 10,000 simple stepwise addition replicates with TBR branch swapping and no more than 10 trees saved per replicate.

Bayesian analysis was performed using NSF Extreme Science & Engineering Discovery Environment (XSEDE) application of MrBayes ver. 3.2.2 (Huelsenbeck and Ronquist 2001; Ronquist and Huelsenbeck 2003) provided by the CIPRES Science Gateway (Miller et al. 2010). PartitionFinder2 was used to test the dataset for partitions (model of evolution: mrbayes; model of selection: AICc; scheme search: greedy) (Guindon et al. 2010; Lanfear et al. 2012, 2016). The best partition scheme suggested six partitions, based on DNA region identity with GTR+G chosen for *matK*, *psbA-trnH*, *trnL-F* and *ycf1* regions and GTR+I+G selected for the *ndhF* and *rbcL* regions. Two independent Metropolis-coupled Markov Chain Monte Carlo (MCMC) analyses were run. Each search used three incrementally heated and one cold Markov chain and was run for 10 million generations and sampled every 1,000^th^ generation. The temperature parameter was set to 0.08. The mean branch length prior was set from the default mean (0.1) to 0.01 (brlenspr = unconstrained: exponential (100.0)) to reduce the likelihood of stochastic entrapment in local tree length optima (Brown et al. 2010). Convergence was assessed using the standard deviation of split frequencies, with values < 0.01 interpreted as indicating good convergence. Tracer ver. 1.6 (Rambaut et al. 2014) was used to determine whether the parameter samples were drawn from a stationary, unimodal distribution and whether adequate effective sample sizes (ESS) for each parameter (ESS > 200) were reached. The first 25% of samples (2,500 trees) were discarded as burn-in and the post-burn-in samples summarised as a 50% majority-rule consensus tree.

### Morphological studies

Comparative morphological data were obtained from specimens deposited in KUN, IBSC and PE herbaria and from published literature. Field surveys were carried out in Menghai County, Yunnan Province, with voucher specimens deposited in HITBC and IBSC.

## Results

The concatenated alignment of the 60-terminal dataset consisted of 7,334 characters. The MP heuristic search retrieved four equally most parsimonious trees of 3,519 steps (consistency index, CI = 0.664; retention index, RI = 0.709).

**Figure 1.**
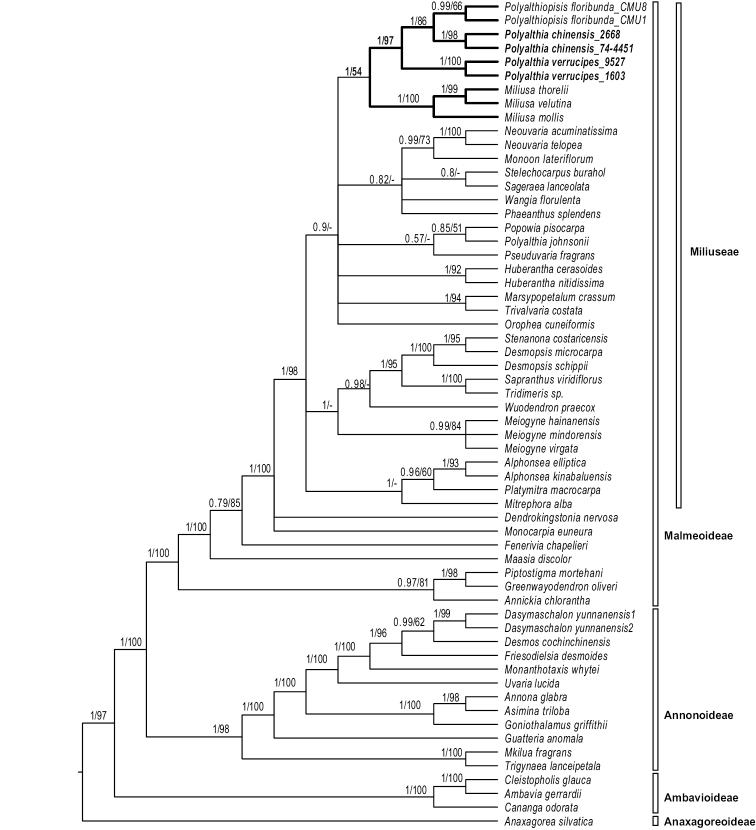
Bayesian 50% majority-rule consensus tree under partitioned models (cpDNA data: *matK*, *ndhF*, *psbA-trnH*, *rbcL* and *trnL-F*; 60 taxa) showing Annonaceae subfams. Anaxagoreoideae, Ambavioideae and Annonoideae. Numbers at the nodes indicate Bayesian posterior probabilities and maximum parsimony bootstrap values (> 50%) in that order.

The MP and Bayesian analyses resulted in similar topologies. The 50% majority-rule consensus tree resulting from the Bayesian analyses under the six partitioned model is shown as Fig. 1. The results are consistent with previous phylogenetic analyses of the family, with the backbone of the tribe Miliuseae unresolved as in previous studies.

*Polyalthia chinensis* and *P. verrucipes* are not retrieved in the same clade as *Polyalthia johnsonii*, but were strongly supported as members of the same clade as *Polyalthiopsis floribunda* (PP [posterior probability] = 1, MPBS = 97%), with the entire clade sister to *Miliusa* with weak to strong support (PP = 1, MPBS = 54%).

## Discussion

*Polyalthia chinensis* was regarded as a synonym of *P. verrucipes* by Hou and Li (2007). The phylogenetic positions of these two species are quite distinct, with the following relationship: (*Polyalthia verrucipes*, (*Polyalthia chinensis*, *Polyalthiopsis floribunda*)) (Fig. 1). Our field collection of the flowers of *Polyalthia verrucipes* provides further evidence for the distinction between these two species.

**Figure 2.**
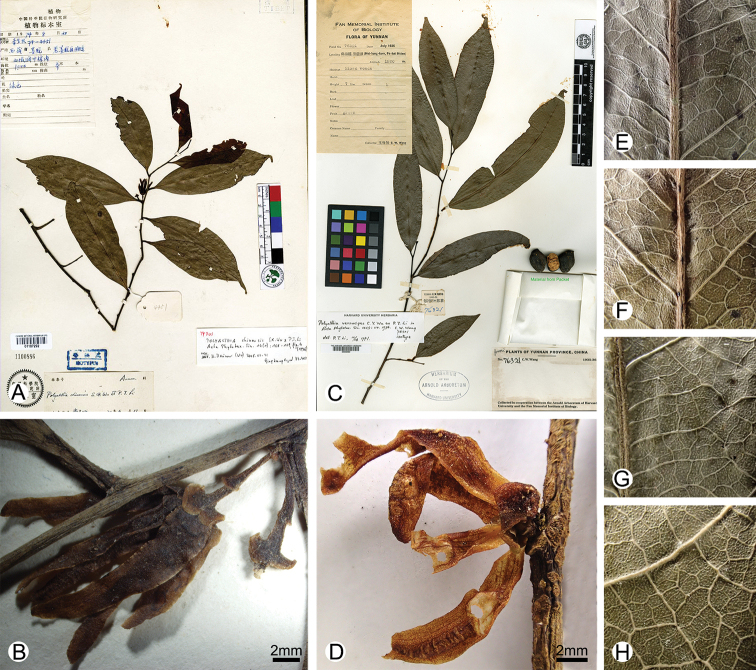
Morphological comparison between *Polyalthiopsis chinensis* and *P. verrucipes***A** type specimen of *P. chinensis* (*Qinghai-Xizang Exped. 74-4451*, PE) **B** infloresence of *P. chinensis* (*Qinghai-Xizang Exped. 74-4451*, PE) **C** type specimen of *P. verrucipes* (*C. W. Wang 76321*, A) **D** inflorescence of *P. verrucipes* (*B. Xue & H.B. Ding 311*, IBSC) **E** adaxial leaf surface of *P. chinensis* (*Qinghai-Xizang Exped. 74-4451*, KUN) **F** abaxial leaf surface of *P. chinensis* (*Qinghai-Xizang Exped. 74-4451*, KUN) **G** adaxial leaf surface of *P. verrucipes* (*Yunnan Exped. 9527*, KUN) **H** abaxial leaf surface of *P. verrucipes* (*Yunnan Exped. 9527*, KUN).

**Figure 3.**
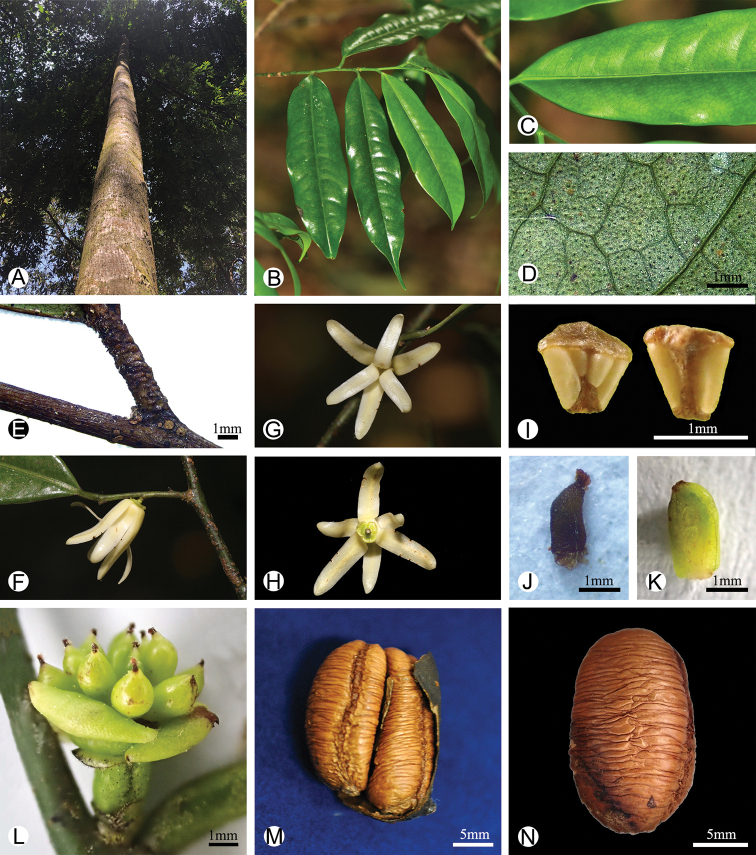
Morphology of *Polyalthiopsis verrucipes* comb. nov. **A** trunk, showing greyish bark **B** a branch, showing the leaf lamina **C** adaxial leaf surface, showing the raised midrib **D** close-up of the abaxial surface of dried leaf, showing glands **E** petiole with transverse striations when dried **F** lateral view of the flower **G** top view of the flower **H** bottom view of the flower **I** adaxial and abaxial view of the stamen **J** carpel **K** longitudinal section of a developing carpel, showing two developing ovules **L** developing young fruits **M** single dried monocarp, showing the two seeds 01187409 (*C. W. Wang 76321*, PE) **N** cylindrical seed, showing longitudinal groove around circumference (*C. W. Wang 76321*, PE). – Photos: **A, C–E, I–N**, by Bine Xue; **B**, by Yun-Hong Tan; **G–H** by Hong-Bo Ding.

Although these two species resemble each other vegetatively (Fig. 2A, C), they differ in the number of flowers per inflorescence, the length and thickness of the pedicel and the colour of the petals. The inflorescences of *Polyalthia chinensis* have 1–2 flowers (Fig. 2A, C), whereas those of *Polyalthia verrucipes* comprise a solitary flower (Figs 2D, 3F, G). The pedicel of *P. chinensis* is slender and ca. 7 mm long, whereas that of *P. verrucipes* is stout and shorter than 2 mm. The petals of *P. chinensis* are green (Li 1976), whereas those of *P. verrucipes* are white (Fig. 3F–H). The leaf also differs slightly, with the leaf lamina of *P. chinensis* (2.5–3.8 cm) narrower than that of *P. verrucipes* (2.5–5 cm) and slightly thinner. The morphological data are therefore congruent with the phylogenetic topology and our phylogenetic and morphological analyses support the hypothesis that both species are not conspecific, as suggested by Li and Gilbert (2011).

The monotypic genus *Polyalthiopsis* Chaowasku was published in 2018, based on *Polyalthia floribunda* collected in Vietnam (Chaowasku et al. 2018). It was reconstructed as the sister group of *Miliusa*, but without statistical support. *Polyalthiopsis*, *Huberantha* and *Miliusa* have previously been retrieved as an unsupported to weakly supported clade in Chaowasku et al. (2018). Although Chaowasku et al. (2018) mentioned that a more comprehensive phylogenetic study, using the whole plastome sequence data, demonstrates the same topology with strong support, the result has yet to be published. *Polyalthiopsis* is also retrieved as sister to *Miliusa* in this study, with weak support in the maximum parsimony analysis (MPBS = 54%), but strong support in the Bayesian analysis (PP = 1). This sister relationship was also well supported in Xue et al. (2020) (ML BS = 86%, suppl. material 1: fig. S1). The relationship between *Huberantha* and the *Polyalthiopsis*-*Miliusa* collective clade is, however, not retrieved in this study. The long-recognised sister relationship between *Miliusa* and *Huberantha* in previous studies (Mols et al. 2008; Saunders et al. 2011; Xue et al. 2011, 2012; Chaowasku et al. 2012, 2014; Chatrou et al. 2012; Guo et al. 2017) can be redefined here following the inclusion of *Polyalthiopsis*.

*Polyalthiopsis* Chaowasku is easily distinguished from most of the other genera in the tribe Miliuseae by its raised midrib on the adaxial leaf surface. When dry, such an adaxial leaf midrib appears slightly sunken. The raised midrib on the adaxial leaf surface is rarely observed in the Annonaceae, but is known from *Artabotrys* (Sinclair 1955; Turner 2012), *Cremastosperma* (Pirie 2005), *Cyathocalyx* (Surveswaran et al. 2010), *Isolona* (Couvreur 2009), *Monodora* (Couvreur 2009), *Mezzettia* (van der Heijden and Kessler 1990), *Pseudephedranthus* (Erkens et al. 2017) and *Stelechocarpus* (Chaowasku et al. 2013; van Heusden 1995). Another distinct feature of *Polyalthiopsis floribunda* is the dried petiole with multiple transverse striations (Chaowasku et al. 2018). Not many Annonaceae species have this pronounced drying artifact. One more distinct feature is the obvious foliar glands on the leaf surface when dried (obvious in fig. 2C in Chaowasku et al. 2018). Foliar glands are also observed in *Wuodendron* B.Xue, Y.H.Tan & Chaowasku in Miliuseae (Xue et al. 2018).

Based on one species with only two collections, the genus is not well described and compared and, hence, it is difficult to identify important diagnostic characters.

**Figure 4.**
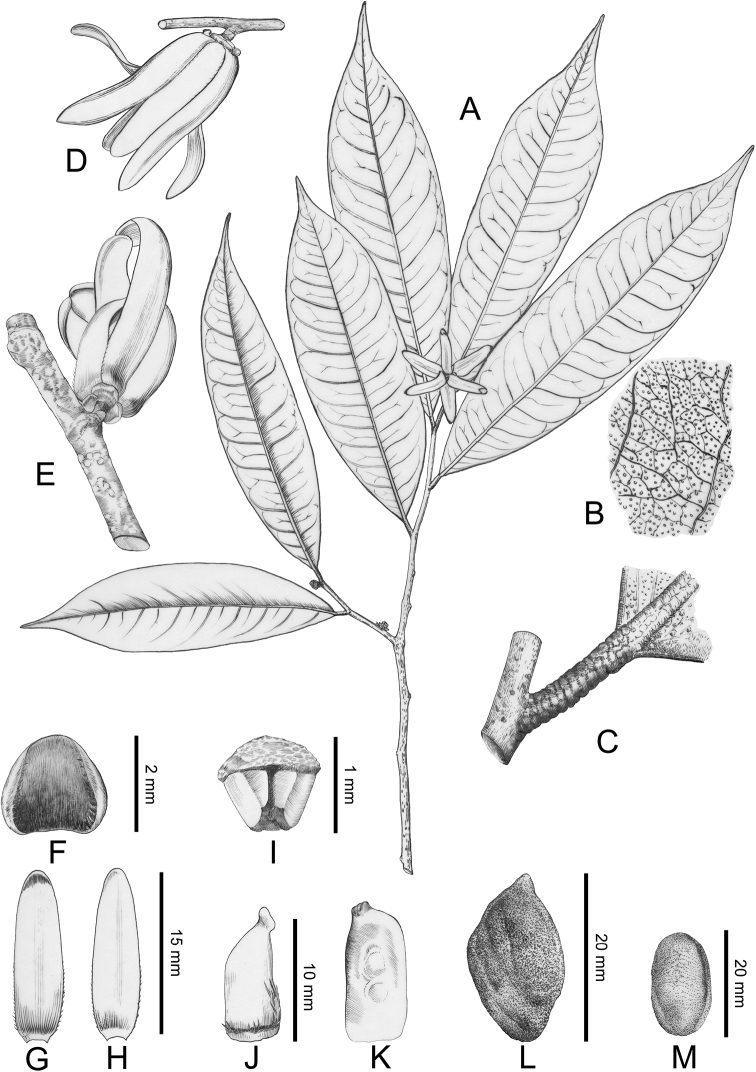
*Polyalthiopsis verrucipes* comb. nov. **A** flowering branch **B** close-up of adaxial surface of leaf, showing glands **C** close-up of leaf petiole, showing the transverse striations on dried petiole **D** lateral view of the flower **E** adaxial view of the flower **F** sepal **G** outer petal **H** inner petal **I** stamen **J** carpel **K** longitudinal section of the developing carpel, showing two lateral ovules **L** a dried monocarp **M** a seed. Drawn by Ding-Han Cui. (**A–K** from *B. Xue* & *H. B. Ding XB311*, IBSC; **L, M** from *C. W. Wang 76321*, PE)

*Polyalthia chinensis* and *P. verrucipes* are retrieved in the same clade as *Polyalthiopsis floribunda* in the molecular phylogeny (Fig. 1). Sterile material of these three species is very similar. The leaves are elliptic with a cuneate base and acute to acuminate apex, with brochidodromous venation and reticulate tertiary veins. The leaf midrib in all three species is furthermore raised adaxially *in vivo* (Fig. 3C; raised midrib still visible in the specimen of *P. chinensis*), with multiple transverse striations on the dried petiole (Figs 3E, 4C) and obvious foliar glands on dried leaf surface (Figs 2E–H, 3D, 4B). Although all three species have axillary inflorescences, the number of flowers per inflorescence differs: *P. chinensis* has one to two flower(s) per inflorescence (Fig. 2A, B), *Polyalthia verrucipes* has only one flower per inflorescence (Figs 2D, 3F–H), while *Polyalthiopsis floribunda* has 1–5 flower(s) per inflorescence. The shape of the petal also differs: the petals of *Polyalthia chinensis* and *P. verrucipes* are linear (Figs 2B, D, 3F–H, 4D, E), while those of *Polyalthiopsis floribunda* are elliptic-ovate (Chaowasku et al. 2018). The carpel characters of *Polyalthia chinensis* and *P. verrucipes* also differ greatly from those of *Polyalthiopsis floribunda*: the former two species have two ovules per ovary and hence two seeds in each monocarp (Fig. 3K, M), whereas *Polyalthiopsis floribunda* has only one ovule per ovary (Jovet-Ast 1940; Chaowasku et al. 2018).

In conclusion, *Polyalthia chinensis*, *P. verrucipes* and *Polyalthiopsis floribunda* share axillary inflorescences, a raised midrib on the adaxial leaf surface (Fig. 3C), petioles with transverse striations when dry (Fig. 3E) and foliar glands on dried leaf surface (Figs 2E–H, 3D, 4B). These characters render the three species distinctive from other species in the tribe and are thus diagnostic for the enlarged genus *Polyalthiopsis*.

The present phylogenetic study shows that *Polyalthia chinensis* is strongly supported as sister to *Polyalthiopsis floribunda* (PP = 1, MPBS = 86%). The collective clade is strongly supported as sister to *Polyalthia verrucipes* (PP = 1, MPBS = 97%). The whole clade (comprising the three species) is weakly to strongly supported (PP = 1, MPBS = 54%) as sister to *Miliusa*. The morphological and molecular phylogenetic data therefore support the transfer of *Polyalthia verrucipes* and *P. chinensis* to *Polyalthiopsis* and the new nomenclatural combinations are proposed here.

As *Polyalthia verrucipes* was published based on fruiting material only (Li 1976), with the newly collected flowers, an updated description is presented. It is noteworthy that the floral description of *P. verrucipes*, published by Li and Gilbert (2011), does not correspond with the material we collected in the field, but is instead similar to that of *P. chinensis*.

As more species were included in the genus *Polyalthiopsis*, an updated description and a key to the three species is also provided.

### Taxonomy

#### Polyalthiopsis

Taxon classificationPlantaeMagnolialesAnnonaceae

Chaowasku, Ann. Bot. Fennici 55: 130. 2018.

959A71D2-149F-5E9B-BA95-45393380B607

Figs 2 , 3 , 4 , 5

##### Type species.

*Polyalthiopsis floribunda* (Jovet-Ast) Chaowasku – *Polyalthia floribunda* Jovet-Ast, Notul. Syst. 9: 75. 1940. – *Huberantha floribunda* (Jovet-Ast) I.M.Turner, Webbia 71: 229. 2016. – Type: Vietnam. Phanrang Prov., Tra Ca, 10 March 1924, *Poilane 10052* (holotype P [barcode no. P00411080]; isotypes A[barcode no. A00351290], BO?, CMUB, HN, K[barcode no. K000608178], L[barcode no. L3728819], P [barcode no. P00411081; P00411082]), in flower.

##### Description.

Medium-sized to large trees. Young twigs glabrous. Leaves petiolate, blade elliptic, with glandular dots observable when dry, base cuneate, apex acute to bluntly (caudate-)acuminate; petiole with transverse striations when dry; upper surface of midrib raised in living plants, becoming slightly sunken when dry, lower surface of midrib raised; secondary veins rather faint in living plants, becoming slightly raised on both sides when dry, leaf venation brochidodromous; tertiary veins reticulate. Flower(s) in 1- to 5-flowered inflorescences, bisexual, pedicellate; inflorescences axillary, peduncle inconspicuous, bracts present. Sepals broadly ovate-triangular. Petals membranous-papyraceous to leathery. Outer petals elliptic-ovate or linear-lanceolate. Inner petals (narrowly) elliptic-ovate or linear-lanceolate. Stamens numerous per flower, connective truncate, covering thecae. Carpels numerous per flower; ovaries with 1 or more line(s) of hairs; stigma terete; ovule(s) 1 or 2 per ovary, sub-basal or lateral. Monocarps oblong to rhomboidal or cylindrical, stipitate, glabrous. Seed(s) 1or 2 per monocarp, cylindrical, surface smooth, raphe broadly sunken and partially slightly raised in middle, endosperm ruminations lamelliform.

##### Distribution.

**Figure 5.**
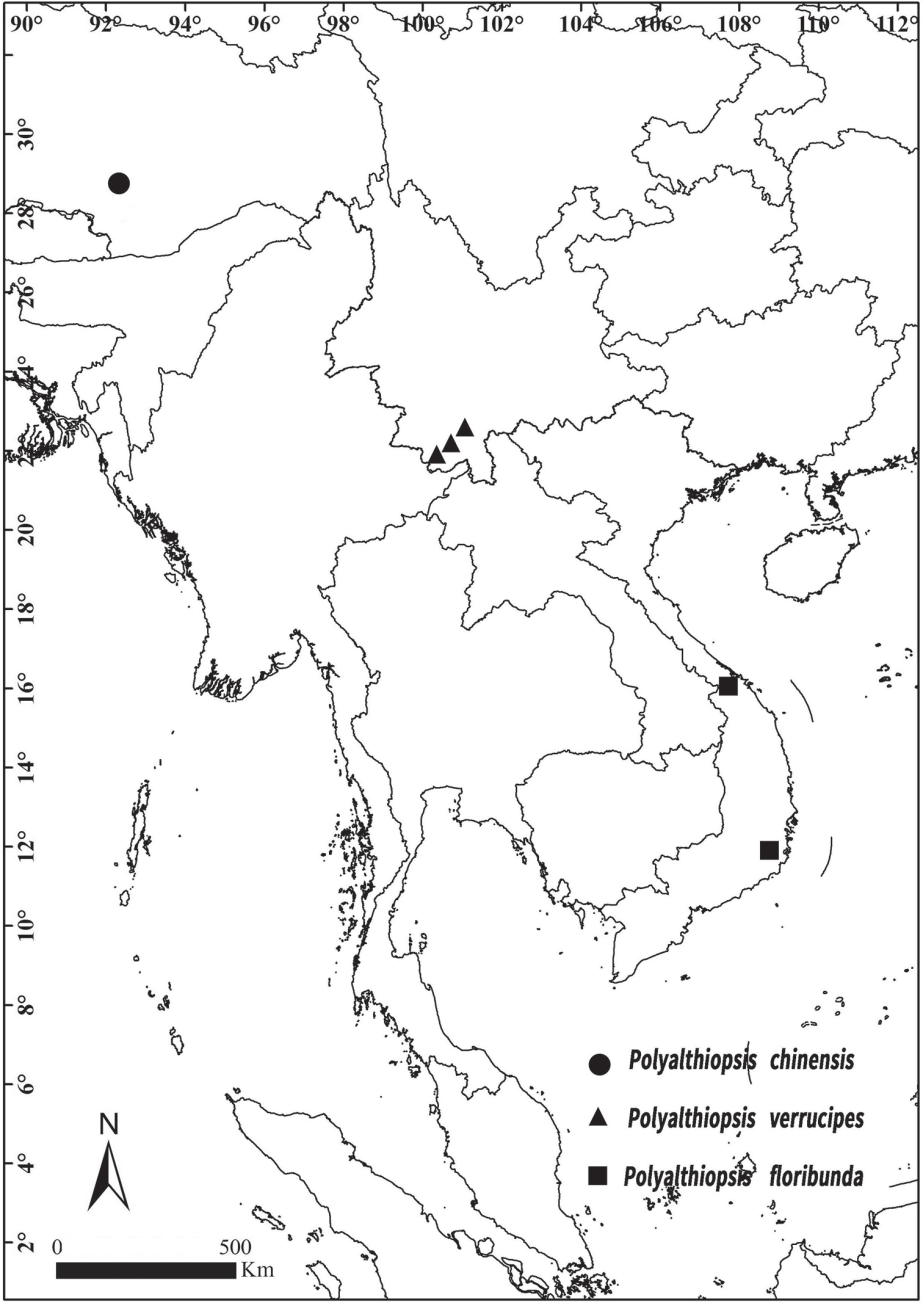
Distribution of *Polyalthopsis chinensis*, *P. floribunda* and *P. verrucipes*.

Three species, known from Xizang, Yunnan Provinces of China and Thừa Thiên-Hu, Ninh Thuận Provinces of Vietnam (Fig. 5).

#### Polyalthiopsis chinensis

Taxon classificationPlantaeMagnolialesAnnonaceae

(S.K.Wu ex P.T.Li) B.Xue & Y.H.Tan comb. nov.

B6164F55-50B2-53DF-9D9D-E7AC47339DDF

urn:lsid:ipni.org:names:77209705-1

Figs 2 , 5

Polyalthia chinensis S.K.Wu & P.T.Li in Acta Phytotax. Sin. 14 (1): 108, t. 4. 1976. Basionym

##### Type.

China. Xizang: Mêdog, 20 Auguest 1974, *Qinghai-Xizang Exped. 74-4451* (holotype, PE! [barcode no., PE01187290]; isotypes, PE! [[barcode no., PE01187291, PE01187292, PE01187293], KUN! [barcode no., KUN0677650]).

##### Distribution and habitat.

Known from Mêdog in Xingzang Province (Fig. 5), growing in rain forests, at low to medium elevations (800–1000 m a.s.l.).

##### Phenology.

Flowering in August.

##### Additional specimens examined.

*B. S. Li & S. Z. Cheng 2668* (PE).

##### Preliminary IUCN conservation status.

DD (IUCN 2012). This species is only represented by two collections in Mêdog in Xizang Province. As Mêdog is not well explored, we tentatively recommend the conservation status as Data Deficient.

#### Polyalthiopsis verrucipes

Taxon classificationPlantaeMagnolialesAnnonaceae

(C.Y.Wu ex P.T.Li) B.Xue & Y.H.Tan comb. nov.

682DD7C5-A379-546E-909A-FCE806978D83

urn:lsid:ipni.org:names:77209706-1

Figs 2 , 3 , 4 , 5

Polyalthia verrucipes C.Y.Wu ex P.T.Li in Acta Phytotax. Sin. 14 (1): 110. 1976. Basionym

##### Type.

***Lectotype* (designated here).** China. Yunnan: Menghai, July 1936, *C. W. Wang 76321* (IBSC! [barcode no. IBSC0003386]; isolectotypes, A [barcode no. A00039580, photo!], IBSC! [barcode no. IBSC0003386], PE! [barcode no. PE01187287, PE01187470], NAS[barcode no. NAS00321991, photo!]).

##### Description.

Trees to 15 m tall (Fig. 3A). Branches greyish-black, glabrous. Petiole 3–7 mm long, 1–2 mm in diameter, glabrous, with transverse striations when dry (Figs 3E, 4C); leaf laminas oblong to oblong-lanceolate, 10–17 × 2.5–5 cm, base broadly cuneate or obtuse, apex acuminate (Figs 3B, 4A), both surfaces glabrous, thinly leathery, densely verrucate with foliar glands when dry (Figs 2G, H, 3D, 4B); upper surface of midrib raised when fresh (Fig. 3C), becoming flat or slightly sunken when dry (Fig. 2G), lower surface of midrib raised; secondary veins 15–18 on each side of midrib, delicate and prominent on both surfaces; tertiary veins reticulate. Inflorescences axillary, with solitary flower (Figs 2D, 3F, H, 4A, D, E). Pedicel 1–2 mm long, hispid, with one ovate bracteole at top, 2–3 ovate bracteoles at base (Fig. 3F, L). Sepals ovate, 2 × 2 mm, slightly reflexed, ciliate (Fig. 4F). Petals 6, valvate, free, in 2 whorls; white, linear, both whorls subequal, ca. 16 × 3–5 mm, thinly leathery, glabrous, slightly ciliate (Figs 3F–H, 4G, H). Stamens 40–50 per flower, ca. 1 mm long (Figs 3I, 4I); connective truncate. Carpels 12–16 per flower, oblong, glabrous; stigma ovoid, puberulent; ovary with 1 or 2 line(s) of hairs (Figs 3J, 4J); ovules 2 per ovary, lateral (Figs 3K, 4K). Fruiting pedicel becoming longer and thicker, 5–7 mm long, ca. 3 mm in diameter; monocarp stipes 7–9 mm long; monocarps oblong to rhomboid, ca. 2 cm long, 1 cm in diameter (Figs 3M, 4M). Seeds 2 per monocarp, yellowish, semi-ellipsoid to ellipsoid, ca. 18 cm long, 8 mm in diameter, endosperm rumination lamelliform (Figs 3M, N, 4M).

##### Distribution and habitat.

Known from several localities in Yunnan Province (Fig. 5): Hei-long-tan and Manxi in Meng-hai County and Kun-man in Meng-yang Town, Jinghong, growing in rain forests, at medium elevations (1300–1800 m a.s.l.).

##### Phenology.

Flowering in February to March; fruiting from April to July.

##### Additional specimens examined.

China. Yunnan: Kun-man, Xiao-meng-yang, 27 April 1957, *Yunnan Exped. 9527* (IBSC, KUN, PE); Man-xi, Menghai, 16 March 2016, *Y.H. Tan MH1603* (HITBC, IBSC); 5 March 2019, *B. Xue & H.B. Ding 311*, *312*, *313* (HITBC, IBSC, KUN).

##### Preliminary IUCN conservation status.

CR A2ac, C2(a)(i) (IUCN 2012). *Polyalthiopsis verrucipes* was assessed as EN A2c by the China Plant Specialist Group (2004). Prior to this study, *P. verrucipes* was only represented in herbaria by two collections from Yunnan, China (two localities, both of which have subsequently been severely deforested). Our field survey in 2016 identified one population with dozens of individuals of dbh ca. 10 cm and dozens of young treelets in Manxi village, Menghai County. We made a second visit to the location in 2019 and found only a few individuals with dbh larger than 10 cm and few treelets. Herbicide had been used in that location. The bark at the bottoms of the tree trunks was damaged. The local farmers appear to clear the forests in this way for tea plantation and it is anticipated that the trees with damaged bark could not survive. We hope additional undocumented sub-populations will be found and protected, although further field investigation is needed to better understand the current status of populations. At present, we recommend that this species be regarded as critically endangered (CR) based on current IUCN Red List Categories and Criteria (IUCN 2012).

### Key to *Polyalthiopsis*

**Table:** 

1a	Inflorescences 1–5-flowered; petals elliptic-ovate; ovule 1 per carpel; distributed in Vietnam	***P. floribunda***
1b	Inflorescences 1–2-flowered; petals linear; ovules 2 per carpel; distributed in China	**2**
2a	Inflorescences 1–2-flowered; pedicel to 5–7 mm long; flowers green; distributed in Xizang, China	***P. chinensis***
2b	Inflorescences with a single flower; pedicel 1–2 mm long; flowers white; distributed in Yunnan, China	***P. verrucipes***

## Supplementary Material

XML Treatment for Polyalthiopsis

XML Treatment for Polyalthiopsis chinensis

XML Treatment for Polyalthiopsis verrucipes

## Appendix 1

Voucher information and GenBank accession numbers for samples used in this study (–, missing data; *, newly generated sequences). Voucher data are given for accessions for which DNA sequences were newly obtained, using the following format: species, origin, voucher and Genbank accession numbers for *matK*, *ndhF*, *rbcL*, *psbA-trnH* and *trnL-F* and *ycf1*. For DNA sequences published in previous studies, voucher information is available from GenBank.

***Alphonsea elliptica*** Hook. f. & Thomson, AY518807, JQ690401, JQ690402, –, AY319078, JQ690403; ***Alphonsea kinabaluensis*** J. Sinclair, AY518811, –, –, AY318968, AY319080, –; ***Ambavia gerrardii*** (Baill.) Le Thomas, AY220435, AY218168, –, –, AY220411(intron)AY220358(spacer), –; ***Anaxagorea silvatica*** R. E. Fr., AY743477, EF179280, –, AY743439, AY743458, –; ***Annickia chlorantha*** (Oliv.) Setten & Maas, AY841393, AY841401, –, AY841594, AY841671, –; ***Annona glabra*** L., DQ125050, EF179281, –, AY841596, AY841673, –; ***Asimina triloba*** (L.) Dunal, AY743479, EF179287, –, AY743441, AY743460, –; ***Cananga odorata*** (Lam.) Hook. f. & Thomson, AY841394, AY841403, –, AY841602, AY841680, –; ***Cleistopholis glauca*** Pierre ex Engl. & Diels, AY841395, AY841404, –, AY841603, AY841681, –; ***Dasymaschalon yunnanense*** (Hu) Bân 1, JQ768560, JQ768598, JQ768639, JQ768680, JQ768720, –; ***Dendrokingstonia nervosa*** (Hook. f. & Thomson) Rauschert, KJ418392, KJ418386, KJ418400, KJ418382, KJ418407, –; ***Desmopsis microcarpa*** R. E. Fr., AY518804, JX544771, AY841461, AY319059, AY319173, JX544758; ***Desmopsis schippii*** Standl., AY518805, JQ723786, –, AY319060, AY319174, –; ***Desmos cochinchinensis*** Lour., JQ768568, JQ768604, –, JQ768688, JQ768728, –; ***Dasymaschalon yunnanense*** (Hu) Bân 2, KF680919, KF680919, –, –, –, –; ***Fenerivia chapelieri*** (Baill.) R. M. K. Saunders, JF810375, JQ723788, –, JF810387, JF810399, –; ***Friesodielsia desmoides*** (Craib) Steenis, JQ768577, JQ768612, –, JQ768696, JQ768738, –; ***Goniothalamus griffithii*** Hook. f. & Thomson, AY743484, EF179296, –, AY743446, AY743465, –; ***Greenwayodendron oliveri*** (Engl.) Verdc., AY743489, AY841408, AY841465, AY743451, AY743470, –; ***Guatteria anomala*** R. E. Fr., AY740913, EF179298, –, AY740962, AY741011, –; ***Hubera cerasoides*** (Roxb.) Chaowasku, AY518854, JQ723810, KF709055, AY319017, AY319131, JQ723950; ***Hubera nitidissima*** (Dunal) Chaowasku, KF682110, KF682116, KF709056, KF682103, KF682105, –; ***Maasia discolor*** (Diels) Mols P. J. A. Kessler & Rogstad, AY518872, AY841416, AY841500, AY319021, AY841584, –; ***Marsypopetalum crassum*** (R. Parker) B. Xue & R. M. K. Saunders, HQ286571, JQ723792, KF709057, HQ286577, HQ286583, JQ723929; ***Meiogyne hainanensis*** (Merr.) Bân, JQ723773, –, –, JQ723860, JQ723913, JQ723936; ***Meiogyne mindorensis*** (Merr.) Heusden, JQ723776, JQ723800, –, JQ723863, JQ723916, JQ723939; ***Meiogyne virgata*** (Blume) Miq., AY518798, JQ723805, JX544784, AY318982, AY319094, JQ723945; ***Miliusa mollis*** Pierre, AY518851, JQ690503, JQ690504, –, AY319102, JQ690505;*Miliusa thorelii* Finet & Gagnep.,AY518846,JQ690519,JQ690520,-,AY319104,JQ690521; ***Miliusa velutina*** (Dunal) Hook. f. & Thomson, AY518847, JQ690536, JQ690537, AY318993, AY319105, JQ690538; ***Mitrephora alba*** Ridl., AY518855, JQ723807, KF709058, AY318994, AY319106, JQ723947; ***Mkilua fragrans*** Verdc., DQ125060, EF179303, –, AY841634, AY841712, –; ***Monanthotaxis whytei*** (Stapf) Verdc., EF179278, EF179304, –, AY841635, AY841713, –; ***Monocarpia euneura*** Miq., AY518865, AY841412, AY841477, AY318998, AY319111, –; ***Monoon lateriflorum*** (Blume) B. Xue & R. M. K. Saunders, JQ723783, JQ723811, KF709060, JQ723870, JQ723923, JQ723951; ***Neo-uvaria parallelivenia*** (Boerl.) H. Okada & K. Ueda, AY518794, –, –, AY319000, AY319113, –; ***Neo-uvaria telopea*** Chaowasku, JX544751, JX544778, JX544791, JX544755, JX544783, JX544766; ***Orophea cuneiformis*** King, KF682112, KF682119, –, –, KF682107, –; ***Phaeanthus splendens*** Miq., AY518864, JX544777, JX544790, JX544754, AY319126, JX544765; ***Piptostigma mortehani*** De Wild., AY743492, AY841415, –, AY743454, AY743473, –; ***Platymitra macrocarpa*** Boerl., AY518812, JQ723809, KF709062, AY319013, AY319127, JQ723949; ***Polyalthia johnsonii*** (F. Muell.) B. Xue & R. M. K. Saunders, JQ723767, JQ723791, KF709063, JQ723854, JQ723907, JQ723928; ***Polyalthiopsis chinensis*** (S.K.Wu ex P.T.Li) B.Xue [= *Polyalthia chinensis* S.K.Wu & P.T.Li, *Polyalthia chinensis*_74-4451], China, Xizang Province, *Qinghai-Xizang Exped. 74-4451* (KUN), MT239203*, –, –, –,MT239213*, –; ***Polyalthiopsis chinensis*** (S.K.Wu ex P.T.Li) B.Xue [= *Polyalthia chinensis* S.K.Wu & P.T.Li, *Polyalthia chinensis*_2668], China, Xizang Province, *B. S. Li & S. Z. Cheng 2668* (PE), MT239201*, –, –, –, MT239211*, –; ***Polyalthiopsis verrucipes*** (C.Y.Wu ex P.T.Li) B.Xue [= *Polyalthia verrucipes* C.Y.Wu ex P.T.Li, *Polyalthia verrucipes*_1603], China, Yunnan Province, *Y.H. Tan MH1603* (IBSC), MT239202*, MT239205*, MT239207*, MT239209*, MT239212*, MT239215*; ***Polyalthiopsis verrucipes*** (C.Y.Wu ex P.T.Li) B.Xue [= *Polyalthia verrucipes* C.Y.Wu ex P.T.Li, *Polyalthia verrucipes*_9527], China, Yunnan Province, *Sino-Russia Exped. 9527* (PE), MT239200*, MT239204*, MT239206*, MT239208*, MT239210*, MT239214*; ***Polyalthiopsis floribunda*** (Jovet-Ast) Chaowasku, *Chaowasku 168* (CMUB), MG264583, MG264588, MG264570, MG264580, MG264575, –; ***Polyalthiopsis floribunda*** (Jovet-Ast) Chaowasku, *Chaowasku 128* (CMUB), MG264585, MG264590, MG264572, –, MG264577, –; ***Popowia pisocarpa*** (Blume) Endl., AY518862, JQ723812, KF709065, AY319044, AY319158, JQ723953; ***Pseuduvaria fragrans*** Y. C. F. Su, JQ723784, JQ723813, XXXXXXXX, JQ723871, JQ723924, JQ723954; ***Sageraea lanceolata*** Miq., AY518799, JX544774, JX544787, AY319050, AY319164, JX544762; ***Sapranthus viridiflorus*** G. E. Schatz, AY743493, AY841422, AY841515, AY319051, AY319165, JQ723955; ***Stelechocarpus burahol*** (Blume) Hook. f. & Thomson, AY518803, JQ723814, KF709067, AY319053, AY319167, JQ723956; ***Stenanona costaricensis*** R. E. Fr., AY518801, JX544772, AY841516, AY319069, AY319183, JX544759; ***Tridimeris* sp**., JX544750, JX544773, JX544786, JX544753, JX544782, JX544761; ***Trigynaea lanceipetala*** D. M. Johnson & N. A. Murray, AY743487, EF179309, –, AY743449, AY743468, –; ***Trivalvaria costata*** (Hook. f. & Thomson) I. M. Turner, HQ286574, JQ723815, XXXXXXXX, HQ286580, HQ286586, JQ723957; ***Uvaria lucida*** Benth. subsp. ***virens*** (N. E. Br.) Verdc., AY238966, EF179310, –, AY238957, EF179319, –; ***Wangia florulenta*** (C. Y. Wu ex P. T. Li) Bine Xue, KX495154, KX495158, KX495156, KX495162, KX495160; ***Wuodendron praecox*** (Hook. f. & Thomson) B.Xue, Y.H.Tan & X.L.Hou, MF687367, MF687369, MF687371, MF687373, MF687375, MF687377.
